# Where does the EU-path on new genomic techniques lead us?

**DOI:** 10.3389/fgeed.2024.1377117

**Published:** 2024-03-14

**Authors:** Finja Bohle, Robin Schneider, Juliane Mundorf, Luise Zühl, Samson Simon, Margret Engelhard

**Affiliations:** Federal Agency for Nature Conservation, Division of Synthetic Biology Assessment, Enforcement of Genetic Engineering Act, Bonn, Germany

**Keywords:** new genomic techniques, NGT plant, GMO, EC proposal, EU regulation, CRISPR-Cas, environmental risk assessment, ERA

## Abstract

Recently, the European Commission (EC) published a regulatory proposal on plants generated with new genomic techniques (NGTs) (5 July 2023). According to this proposal, NGT plant applications are categorized into category 1 NGT (NGT1) and category 2 NGT (NGT2) based on their molecular characteristics, which diverges from the current legislation centered around Directive 2001/18/EC. To demonstrate where the path of the proposal leads to in practice, we applied the proposed criteria for categorization to a list of NGT plant applications currently in the commercialization pipeline. Combining literature research and a descriptive statistical approach, we can show that 94% of the plant applications affected by the EC proposal, would be classified as NGT1 and thus would receive market approval without risk assessment, monitoring, and sufficient labeling provisions. The remaining 6% of applications would be classified as NGT2 plants, for which, in deviation from the current regulation, an adapted risk assessment is proposed. Screening of the intended traits in the pipeline highlights that certain NGT1 plants can pose similar environmental risks (e.g., invasiveness) to other genetically modified organisms (GMOs), as defined in Directive 2001/18/EC. For example, NGT1 applications based on RNA interference technology can exhibit insecticidal effects with potential side effects on non-target organisms (i.e., other insects). Our quantitative and case-specific elaboration of how the current EC regulatory proposal would affect the environment, health, and consumer protection will be informative for decision-makers and politicians.

## Introduction

In the European Union (EU), deliberate release of genetically modified organisms (GMOs) into the environment and functioning of the market for the corresponding GMO-derived products are regulated by the current Directive 2001/18/EC and Regulation (EC) No 1829/2003. This EU GMO framework has established a prior authorization system that comprises a case-by-case assessment of the risks to human health and the environment associated with releasing GMOs, in accordance with the precautionary principle. Authorization is also linked to mandatory post-market monitoring requirements.

Since Directive 2001/18/EC came into force, development of new genomic techniques (NGTs), including genome editing using CRISPR-Cas, has advanced the genetic modification of plants. With the targeted genetic approach and potential absence of transgenic DNA sequences in the final NGT product, whether NGTs qualify for exemption from the current Directive 2001/18/EC remains under debate. In July 2018, the Grand Chamber of the European Court of Justice (ECJ) ruled that environmental and health risks associated with plants generated by NGTs were comparable to the risks associated with the production and distribution of GMOs generated by transgenesis (European Court of Justice, Confédération Paysanne, 2018). Furthermore, NGTs enable the creation of various GMOs at a much greater pace and scale than random mutagenesis techniques, which, according to the ECJ, argues for strict application of the precautionary principle. Therefore, the ECJ confirmed that Directive 2001/18/EC is applicable to NGTs without restrictions.

Recently, the European Commission (EC) published a regulatory proposal (EC proposal; European Parliament, Council of the European Union, 2023a; European Parliament, Council of the European Union, 2023b) to adapt the prevailing application procedure exclusively to genetically modified (GM) plants, including non-crops generated with NGTs. The EC proposal is accompanied by a whole set of reports (European Commission, 2023b incl. work cited therein, especially by EFSA) and specifically concerns targeted mutagenesis and cisgenesis, including intragenesis, of NGTs in plants. A prerequisite for any NGT application is that no DNA from outside the breeder’s gene pool (non-crossable species) is present in the final NGT plant, including the genetic material that has been temporarily inserted during the technical development of the plant. According to the current EC proposal, NGT plants should be further divided into two categories, based on their genetic modifications defined in Annex I. Category 1 NGT plants (NGT1) shall comprise a maximum of 20 genetic modifications fulfilling the following specifications: (i) deletions or inversions of any number of nucleotides, as well as insertions or substitutions of DNA sequences with up to 20 arbitrary nucleotides, shall be possible anywhere in the genome, and (ii) insertions or substitutions of any-sized contiguous DNA sequences must originate from the breeders’ gene pool and shall not disrupt any endogenous genes. (iii) On the basis that the resulting DNA sequence already occurs in a species from the breeder’s gene pool, any other targeted modification of any size is allowed. All other non-transgenic NGT plants that exceed the NGT1 criteria are defined as category 2 plants (NGT2). To simplify the application procedure, the new EC proposal considers NGT1 plants to be equivalent to conventionally bred plants, and suggests a technical confirmation process without a case-by-case risk assessment and the non-application of all European laws on genetic engineering. In category 1, no approval procedure, no risk assessment, no provision of detection methods, insufficient labeling, and no monitoring is envisaged. For NGT2, hazard identification information shall be required if specific traits and intended use lead to a plausible risk hypothesis. Category 2 would impose reduced requirements for risk assessment, detection, and monitoring. Only classical transgenic plant generated using NGTs would continue to fall under current genetic engineering legislation.

With this proposal, the EU is confronted with a fundamental path decision that affects European goals and standards in terms of climate and nature protection, precautions, and freedom of choice. Currently, two main lines of argument can be described: NGTs and their risk profiles are (i) GMOs under European law, as ruled by the Grand Chamber of the ECJ, or (ii) they should be compared with conventional breeding, as proposed by the recent EC proposal. The diverging reasoning results in different positions on (i) whether NGT plants should remain under the current Directive 2001/18/EC or (ii) whether they should be exempt from it in the future. In view of this discussion, we investigated the NGT plant applications that might be affected by the current EC proposal. We focus on the potential environmental impacts from our perspective as GMO risk assessors, whereas other aspects such as consumer protection, coexistence with organic farming, and patent issues are not addressed in this study.

Here, we show that most NGT plant applications affected by the EC proposal would be regulated as NGT1 (94%). Those 94% comprise a wide variety of crop species and affect many different traits, the most prominent being consumer- and industry-oriented traits. Such NGT1 plants would enter the market without risk assessment, although our analysis suggests that they could bear environmental risks comparable to those of other GMOs, including potential insecticidal NGT1-plants based on RNA interference (RNAi) technology.

### 94% of the affected NGT plants would enter the EU market without risk assessment

**FIGURE 1:**
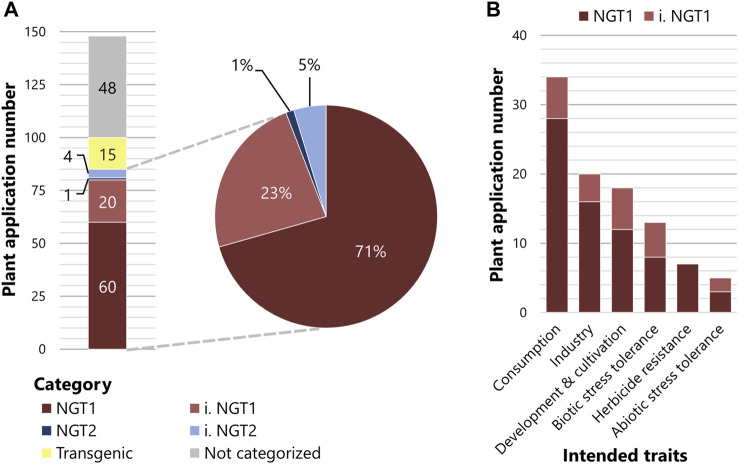
NGT categorization of plant applications listed in Gelinsky (2022). **(A)** The 148 plant applications listed in Gelinsky (2022) were categorized according to Annex I of the European Commission regulatory proposal. Transgenic plant applications are depicted in yellow and plant applications not categorized due to limited information in grey. Of the remaining 85 plant applications, plant applications are either directly categorized into NGT1 (dark red) or NGT2 (dark blue) or inferred to be categorized into NGT1 (i. NGT1, light red) or inferred NGT2 (i. NGT2, light blue). **(B)** Intended trait groups sorted by the abundance of associated plant applications that are categorized to NGT1 (dark red) or inferred NGT1 (i. NGT1, light red). Plant applications with multiple intended traits are sorted to more than one trait group.

To examine where the EC’s proposed path leads us and what the future regulation of NGT plant applications might look like, we analyzed published data on plant applications that are currently in the commercialization pipeline (Supplementary Methods). Our analysis is based on the ‘plant breeding commercialization pipeline and licensing agreements’ list, which was commissioned by the Swiss Federal Office for the Environment (Gelinsky, 2022). We categorized all 148 NGT plant applications according to the NGT1 and NGT2 specifications defined by the EC proposal (Supplementary Table S1). Of these 148 NGT plant applications, 15 would be treated as transgenic NGTs, falling under the current GMO legislation, and 48 plant applications could not be categorized due to a lack of information or data inaccessibility related to confidential business information (Figure 1A). Of the remaining 85 NGT plant applications, 60 could be clearly categorized as NGT1, whereas 20 were assigned to the inferred NGT1 (i. NGT1) (Figure 1A). Only one plant application was categorized as NGT2, and four plant applications as inferred NGT2 (i. NGT2) (Figure 1A). Importantly, of the 85 plant applications affected by the EC proposal, 94% would be classified as NGT1 and 6% as NGT2 (Figure 1A). When evaluated under the scope of the recent EC proposal, a similar distribution of NGT1 and NGT2 plant applications for cultivation and marketing was observed in the United States (U.S.) (Supplementary Table S2), which was selected as a representative non-EU country.

With the development of NGTs, numerous plant species have become accessible for targeted mutagenesis. Accordingly, NGT1 categorized plant applications described by Gelinsky (2022) could be assigned to 26 different plant species (Supplementary Table S1). These include crops grown worldwide on a large scale, such as soybeans (17 plant applications), corn (10), and potatoes (9), and minor crops, such as strawberries (2), raspberries (1), and physalis (1). However, not only crops will be affected by the current EC proposal, but also wild plant species, including trees that can be categorized as NGT1, such as tall fescue, switchgrass, or tree tobacco (Supplementary Table S2).

Plant applications categorized as NGT1 comprised a wide array of postulated traits (Figure 1B; Supplementary Table S1). To visualize this broad range of postulated traits, we divided the NGT plant applications listed by Gelinsky (2022) into six trait groups (a-f) in order of abundance.

(a) Consumption-oriented traits represent the largest group of intended traits (34 NGT1 plant applications), which include traits affecting nutrient content, visual and olfactory modifications, and secondary metabolites of the crop. Examples of visual and olfactory modifications include non-browning of fruits and vegetables.
(b) The second largest trait group includes industry-oriented traits (20 NGT1 plant applications), such as modified ingredient composition, storage and transportability qualities, and bioenergy usage. Camelina plants that have adapted fatty-acid biosynthesis to produce biofuels and dietary supplements represent common examples.
(c) The third group includes traits associated with plant development and cultivation (19 NGT1 plant applications), including plant growth, yield, reproduction, and harvesting. A plant application of this group is a “shatter-tolerant” NGT1 rapeseed developing more stable pods to prevent seed loss during harvesting.
(d) The fourth trait group contains traits that aim to confer tolerance against biotic stressors such as bacteria, fungi, nematodes or viruses (13 NGT1 plant applications). One example is the wheat plant with fungal resistance caused by a mutation that affects plant immune response.
(e) The fifth trait group includes herbicide resistance (seven NGT1 plant applications), which is mainly generated *via* point mutations, e.g., in the *ALS* genes of soybean and rapeseed.
(f) The least represented trait group covers abiotic stress tolerance (five NGT1 plant applications), of which drought tolerance is a favored trait that is proposed to confer adaptation to climate change (Sami et al., 2021; Eckardt et al., 2023).

### Intended traits and unintended risks

The EC proposes simplifying the application process by exempting case-by-case risk assessments for NGT1 plants. Based on our role as GMO risk assessors, we examined whether the potential environmental impact of NGT1 plant applications is comparable to that of GMOs. Therefore, we analyzed some examples mentioned above for the respective trait groups (a-f) and assessed these modified plants regarding the environmental risk areas defined in the respective Directive under which the EC proposal would act (Directive 2001/18/EC).

(i) Persistence and invasiveness: Stress tolerance, both biotic and abiotic, which alters plant fitness may affect plant persistence and establishment in the environment, even for crop plants that are not yet invasive, especially in changing climate regimes. The NGT1 plant applications listed by Gelinsky (2022) comprise a potentially invasive tree tobacco that showed increased fitness after drought stress (Negin et al., 2023). Such drought stress-tolerant tobacco could potentially grow in areas that had previously been too dry and thus might lead to risks for biodiversity in the corresponding ecosystems. Generally, the risk of generating plants with increased persistence and invasiveness might be enhanced when widespread plants such as wild grasses, trees, and herbs become targets of genetic modification, as listed in the U.S. APHIS plant applications (Blackburn et al., 2019; Supplementary Table S2). However, the EC proposes not to require any monitoring or detection concepts for NGT1, which restricts risk management, including the capacity to remove invasive NGT plants and protect biodiversity.
(ii) Gene transfer and selective disadvantages: The transfer of traits from domesticated plants to wild plant species can result in altered weed spread and establishment of novel weeds, which can lead to an increased risk of the extinction of wild species (Ellstrand, 2003). We identified a NGT1 rapeseed, which grows more stable pods to prevent seed loss during the harvesting process (“shatter-tolerance”, Supplementary Table S1). Unintended crossing of the “shatter-tolerant” NGT1 rapeseed with wild plant populations could affect the fitness of wild plants and their natural reproduction due to possible restrictions in seed dispersal.
(iii) Altering cultivation, management, or harvesting techniques: GMOs must be analyzed with regard to their impact on cultivation, management, and harvesting techniques compared to non-GMO plants. This includes a potential increase in insecticide, herbicide, or pesticide usage. We identified non-browning fruits and vegetables that could affect the cultivation system. The “non-browning” trait often comprises a mutation in at least one polyphenol oxidase gene. Polyphenol oxidases play a role in plant pathogen defense, and their loss is associated with impaired biotic stress responses (Thipyapong et al., 2004). A modification of the plant’s pathogen defense mechanism might change the plant’s susceptibility to biotic stress and therefore might alter plant pest management by a potential increase in pesticide usage.
(iv) Interactions with target and non-target organisms (NTOs): Adverse environmental effects resulting from direct and indirect interactions between GM plants and NTOs can also be identified in NGT plants. An example in which adverse interactions with NTOs cannot be excluded is a patented NGT1 plant application. In this case a NGT1 plant would carry a genome edited microRNA (miRNA) conferring insecticidal activities in target (and potentially non-target) insects (see section “Within the realms of possibility: The NGT1-RNAi case”). In other cases, changes in metabolomics, such as protein or lipid content and composition, as seen for many NGT1 plant applications by Gelinsky (2022), may also unintentionally affect the synthesis of by-products and secondary metabolites that are potentially harmful to NTOs (Kawall, 2021).

These examples demonstrate that NGT1 plants can pose direct or indirect risks to human health and the environment, as specified by Directive 2001/18/EC. Importantly, such NGT1 plants would not be risk-assessed according to the EC proposal, and potential hazards would thus not be recognized and evaluated in advance of NGT1 plant release. We conclude that categorization according to molecular parameters, as suggested in the EC proposal, would not exclude risks, and consequently, would not *per se* define plants without risks.

### Within the realms of possibility: The NGT1-RNAi case

**FIGURE 2:**
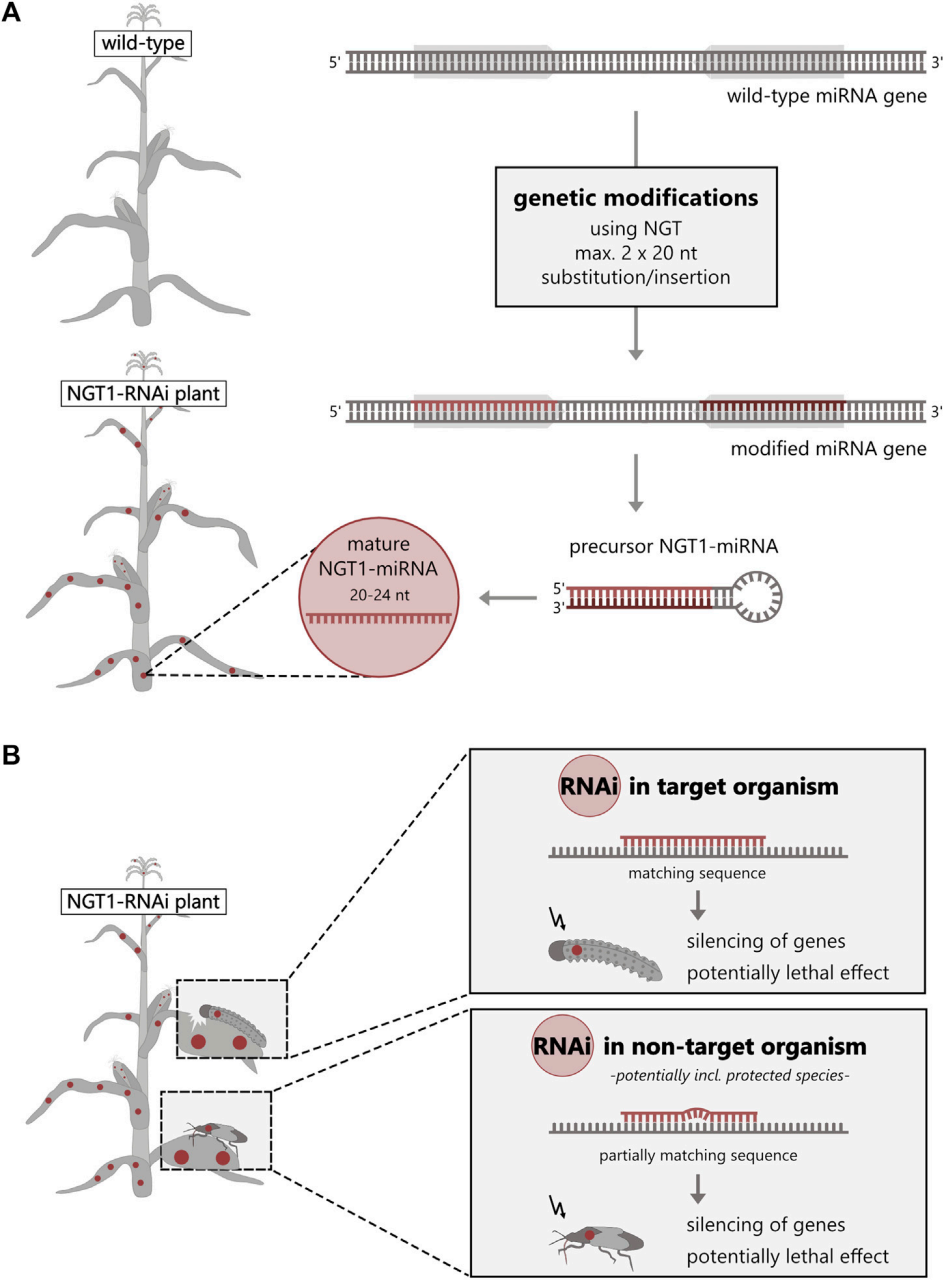
NGT1-RNAi plants can silence genes in other organisms. **(A)** NGT1-RNAi plants can be generated by genetically modifying (red) the recognition sequence [usually 20–24 nucleotides (nt)] of an endogenous miRNA gene (grey arrows) of a wild-type plant. For this, substitution/insertion of a maximum of 20 nucleotides at two positions is sufficient. **(B)** The NGT1-RNAi plant forms an effective, mature NGT1-miRNA from the modified NGT1-miRNA gene by a multi-step cellular RNAi processing mechanism. The precursor (hairpin structure) and/or effective, mature NGT1-miRNA are taken up (orally) by the target and non-target organisms. By triggering the endogenous cellular RNAi machinery, this induces silencing of transcripts (mRNA) of target and potential off-target genes with potentially lethal effect.

NGTs can be used to genetically modify the plant’s own microRNA (miRNA), enabling the silencing of essential proteins in target and non-target insects by so called RNAi. Among the analyzed plant applications, we identified a NGT1 plant application that utilized this molecular mechanism of RNAi to generate non-browning potatoes (Supplementary Table S1). RNAi is an endogenous cellular mechanism that regulates gene expression in most eukaryotes including plants (Shabalina and Koonin, 2008). Here, we describe for the first time the possibility of generating a fully functional, miRNA-based RNAi application (Yu et al., 2017; Song et al., 2019) that would be classified as NGT1 according to the EC proposal (Figure 2). This method has already been patented in the EU and is known as “Gene Editing induced Gene Silencing” technology (GEiGS^®^) (Maori et al., 2019). Among others, Maori et al. (2019) included a potentially insecticidal maize in their patent. In this maize, the endogenous miRNA (*zma-MIR166h*) would be redirected against an essential gene transcript of chitinase in the European corn borer by modifying 20 nucleotides of the miRNA gene at the critical miRNA sequence (Maori et al., 2019) (similar to Figure 2A). The oral uptake of such NGT1 miRNA-expressing maize is assumed to have a lethal effect on the target species (Arakane and Muthukrishnan, 2010; Khajuria et al., 2010; Lu et al., 2023) with potential risks for NTOs, potentially also including protected species (Figure 2B). In this case, the environmental risks are comparable to those of transgenic plants expressing insecticidal RNAi constructs or toxins derived from *Bacillus thuringiensis* in so-called “Bt crops”. Although they share the same principal mechanism as other RNAi applications, such as dsRNA sprays or transgenic GMO (Liu et al., 2020), NGT1-RNAi applications would not be risk-assessed under the current EC proposal, even though this is a strict requirement for transgenic RNAi plants (regulated by Directive 2001/18/EC) or RNAi spray applications (regulated by EC No. 1107/2009 and Directives 283/2013 and 284/2013).

## Discussion

Based on published data on NGT plants in development or undergoing commercialization (Gelinsky, 2022), we show for the first time that the EC’s proposed path would, in fact, lead to deregulation of 94% of these affected NGT plants, as they would fall under category 1. Therefore, they would receive market approval without risk assessment, monitoring provisions, or the need for providing detection methods. When analyzing selected examples of NGT1 plant applications in the commercialization pipeline, we identified possible risks to the environment and human health, which are comparable to the risks of classical GMOs. For example, RNAi applications in plants have the potential to severely affect NTOs, including protected species. Several RNAi plant applications have already been approved as GMO applications in the EU and other countries (European Commission, 2015a; 2015b; 2019; 2023a) and have a growing market perspective (Hernández-Soto and Chacón-Cerdas, 2021; Koch and Wassenegger, 2021).

Generally, the proposal would most likely lead to the increased authorization of NGT plants (RNAi-based and others), for which justified concerns in the sense of the precautionary principle exist. Importantly, the regulatory proposal does not foresee any instrument to retrieve authorization for NGT1 plants, even in cases where a hazard might be shown after release. Our data showed that already today, NGT2 plants represented only a small fraction of the NGT plant applications. However, an even broader spectrum of NGT plant applications may be expected in the future, as already observed in the U.S. (Supplementary Table S2). This is on the one hand owed to the rapid development in the field of genetic engineering. On the other hand, the EC proposal for NGT1 plants might also act as an incentive to design new plant varieties that fulfill the criteria for NGT1. This incentive effect would lead to more NGT1 plant applications entering the market without an environmental risk assessment.

We observed an increasing number of modified crop species, including crops that had not reached the EU market as transgenics in the past (e.g., strawberries and physalis). For NGT plant applications, we also observed diversification of traits compared to the dominant traits for transgenic GMOs (insecticide resistance and herbicide tolerance). In contrast to general expectations, we observed quantitatively more NGT1 plant applications in the prevailing trait groups that are consumption- and industry-oriented when compared to a minority of the expected traits that are supposed to enable, for example, adaptation to climate change (abiotic stress).

As discussed above, we show that, most likely, for all future NGT applications the current GMO framework would not apply, which includes risk assessment and labeling, among others. Our analysis also reveals, that current NGT1 applications can clearly pose potential risks in both relevant areas, the environment and human health, according to Directive 2001/18/EC (Annex II D.2). Therefore, it can be concluded that the presumed equivalency with conventional plants, defined by Annex I of the proposal, is not a suitable criterion for assuming the safety of NGT plant applications. In contrast, proof that these applications pose fewer risks than other genetic engineering products (i.e., transgenics) is inevitable. Here, we demonstrate that this cannot be assumed for current NGT1 cases. Furthermore, the specific criteria proposed in Annex I of the EC proposal are unsuitable for proving equivalence to conventionally bred plants: We show concrete examples of NGT1 plant applications, which clearly cannot be produced with conventional breeding tools, as the NGT1-RNAi case demonstrates particularly impressively. With respect to risk, we will be unable to avoid considering additional denominators for the profound assessment of NGT plant applications (Eckerstorfer et al., 2021).

With the current EC proposal and associated political negotiations, the EU is now at a crossroad, where decisions on NGTs will have a far-reaching impact on the environment, land usage, and biodiversity. Acknowledging the precautionary principle, the EU should responsibly decide on the path it will take in the future.

## Data Availability

The original contributions presented in the study are included in the article/Supplementary Material, further inquiries can be directed to the corresponding author.
